# Retiring Address*Published in Texas Medical Journal by vote of Society.

**Published:** 1905-09

**Authors:** C. M. Harrison

**Affiliations:** Cooper, Texas; President Delta County Medical Society


### Retiring Address.*

BY C. M. HARRISON, M. D., COOPER, TEXAS.
President Delta County Medical Society.

This is our annual meeting—the business meeting of the society under the new regimé. We have now been working more than a year under the reconstruction, and, like all good business men, we should "take stock." At least find out what are our assets and liabilities. When a doctor permits his liabilities to exceed his assets, humanity is in danger.

I am happy to be able to say to you that the condition of this society is quite a little better than before our reorganization; that the work done by its members has been better than before and of a higher grade; but still, it isn't nearly what it should be. Possibly there are some who think they can learn nothing by attending; if so, they should, like true physicians, come and impart some of their medical lore to their associates, thus helping along the work as duty and honor demands.

There are quite a considerable number of the physicians of the county who have not become members. I can conceive of no reason why a physician of good repute, and who has the interest of his profession at heart, can refuse; especially so, when there is scarcely any expense, and requires so little of his time. Evidently he doesn't know that the secretary is required to furnish the Secretary of the State Association the names of all the non-members, whether or not they are graduates, ethical or unethical; in short, "keep tab" on them; and that business corporations seeking a

*Published in TEXAS MEDICAL JOURNAL by vote of Society.

physician would look askance at a name not on the roster of his county society. It is to be hoped that within the next year we will be able to enroll all the physicians of our county; and that the older members will shake off their lethargy and "get busy" in a common interest.

This appears to me to be an imperative duty of every member. The county societies are the gateways, the only doors to the State Association, and to the American Medical Association; and the day is not far distant when it will be considered irregular not to belong to them. Just as the units, so is the composite number. So will the State Association reflect the county societies.

It is true that our present organization is new, practically untried, and I have no doubt that the motives of its promoters are unselfish and pure; at the same time, its potentialities, if abused, are illimitable and dangerous. The conception of making the county societies the unit of organization, and placing the censorship of professional standing in the hands of the medical commoner, is the product of a deep philosophy. It comports with American ideas and principles, recognizes the rights of the professional constituency—the working class, instead of leaving it, as it has been largely done heretofore, in the hands of that twentieth century abomination, the medical politician.

From my view-point this is the explanation of the success and growth of the new organization, and accounts largely for the eagerness and loyalty of the large numbers of professional men who have responded so willingly.

There is little doubt that the profound indifference with which the old State association was regarded by the great horde of medical men throughout the State was the result of the mismanagement by those who were connected with it; who seemed to regard the rank and file of the profession too patronizingly; knowing, as every one knows, that it is the "man with the hoe" who furnishes the foundation of the superstructure that attests the culmination of human efforts. We all know that heretofore our State association has been practically a failure. If it succeeds in the future, if our new organization is to endure, the county societies must make it so. Upon them rests its growth or decay. The portals must be well guarded, its dignity established and maintained, its members made to know that it is no toy; those who aspire to high places that they must labor with honor, adding luster to renown rather than display their wares.

It is here, again, that the county societies must be the chastening hand, the training school for those with altitudinous visions. Here it is that purity of professional morals must be inculcated; the physician imbued with immaculate principles of professional integrity; those exalted ideas that elevate him above the "common plane of groveling mankind, higher than Hercules above giants, Socrates above philosophers, Solomon above kings"; in a word, that his vocation is for the uplifting of humanity, and not selfish commercialism.

Don't understand that I would repudiate the financial side of our work; on the contrary, I would urge better and more efficient methods, but they should be formulated in a way commensurate with the dignity of our profession without stooping to the tricks of trade.

I believe that it should be unprofessional for a physician to advertise in any way than to put his name plate at his office and residence, and simple letter heads. Our "principles of ethics" are very indefinite on this point; with our commercial spirit and human nature so predominant, the temptation so great, we often wink at little irregularities that are purely commercial in motive, unethical in principle; so that, in the absence of specific law, many take the advantage.

One prominent writer, advising young physicians, recommends that they identify themselves with some church or lodge as a means of bringing themselves into public notice. Such advice is pernicious. No one who is worthy to bear our standard will stoop to prostitute a noble institution that he may gratify his personal ambition; nor will he besmirch the fair name of his vocation by resorting to such unmanly tactics.

It is equally as nauseating to permit preachers and newspaper men to go around prating about one's superiority, or to call attention to one's practice or operations. They do the same thing about Peruna and Liquozone, etc., and on such subjects they are totally incompetent to give an opinion.

Also it should not be forgotten that other physicians hearing them, know all about the ability of those of whom they prate; and it always makes one think of a patent medicine show, or the picture of some quack in the daily paper advertising the cure of lost manhood.

To permit political or church meetings in one's life, announcing it publicly beforehand, is not very far removed from charlatanism.

Contract practice, work for the county, city, or any other public business or corporation at a less price than for the humblest taxpayer, is irregular practice, and should be condemned in the strongest terms.

Stocking up an office with X-ray machines, hot air apparatus and other paraphernalia of the grafter, then announcing as a specialist, when only a general practitioner of ordinary cult, is quite sufficient to give a decent man cholera morbus.

Nothing, in my opinion, is more compromising in a physician than to write an article for a journal, and send his photograph along, to be put at the head of it, like a testimonial in an almanac for Thedford's Black Draught. It is difficult to tell whether he is suffering from atrophia cerebri or acute hypercholia. One may write all he wishes for publication and sign his name to it; the critics will take care of that; but he should, at least, have respect enough for his medical friends than to adopt the methods of a street crier. This, also, applies to medical journalism. If I were going to publish a medical almanac, I would do it, and reap the emoluments; but if I were going to publish a journal for an honorable professional constituency, I would try to not get it mixed up with the almanac business.

It has always appeared to me to be rather straining on the proprieties of medical ethics for a practicing physician to engage in the drug business, and suffer his name to be posted on every tree, stump, log, barn or bridge in the country to attract the attention of every passer-by.

But all the evils are not confined to the rural districts. They are found in abundance where one would think the atmosphere pure, their enormity usually being commensurate with the locality. Most prominent among them is the medical college infamy. Texas has eight medical colleges (Heaven spare the name), one of them being a negro college.

Every one knows that there is no dearth of doctors in this State; we have carloads to spare. Neither is there such an ebullition of medical lore among our M. D.'s as to require an escape valve for their erudition; nor have I observed among them such a gallactorrhea of the milk of human kindness as to necessitate the annual drafting of several hundred students to drink up the surplus pap. One can not but be impressed that such motives are mercenary, the cultured germ of greed and power. When the American Eagle tore the British ensign from its flagstaff at Yorktown, it gave us a degree of liberty susceptible of abuse beyond the application of law; nor have the medical college crank, or the professional aspirant failed to find the gap; neither have they hesitated to take the advantage. But there is a higher law, a moral code that underlies all law that is yet supreme, before whose mandates the trickster must bow, and feel the calumny of an indignant and outraged profession.

That every church that is able to maintain a literary school should deem it proper and right to have a medical annex, is nothing short of a selfish disregard for the great humanitarian efforts of the profession,—an unwarranted means of gaining rank and prestige,—clothed in the habilaments of the Great Physician, foisting upon the uninformed inferior institutions that do not educate. It is a reflection upon the Christian purity of the church, a disgrace to the medical profession that it should grant such latitude to its otherwise worthy members. Human life is too sacred to be dealt with as a commercial commodity. A reaction is sure to come. It is proper and right that every State should have its own medical school. This we have in the medical department of the State University. Don't think, for once, that I think that the school at Galveston is all, or nearly all, that it should be; I do not; yet one medical school in a State is enough for all necessary and legitimate demands; this one, liberally endowed, as the Galveston school is—a faculty commensurate with our broad interests—the staunch moral support of the entire profession—it would be something of which every Texan would be proud.

Contrast this with a college existing largely upon paper, recently paraded before a gullible, yet influential, public,, greedy for power and expansion; then several other schools stuck around in the forks of the creeks, street crossings and cross-roads, one of which, the first year after our new law went into effect, stated in its announcement catalogue, that it stood in close touch with the medical board, etc.—a total disregard of the people, of the committee that framed the law, an insult to the legislative body that passed it, and a very serious reflection upon the integrity of the State examining board. If the board ever resented, I never heard of it. Think of it, gentlemen, and if your manly spirit doesn't rebel at such high-handed grossness, you are not made of the material that I think that a medical man should be. I ask, don't you think Texas needs a law regulating medical colleges as badly as she needs one regulating the practice? We have regulated the latter, in a measure, and have let the other go on without "rein or drag." Statesmanship, American citizenship, duty and honor, our own professional integrity, all demand that such gross violence to decency be eliminated, and the initiative must of necessity be taken in the county society.

I wish now to call your attention to the patent medicine curse. Our laws have not yet reached these gentry. To require them to put their formula on the bottle is a very small requirement. They should be compelled to walk up to the lick log of the examining board and take their medicine like you and me. Recently a couple of counter-jumping clerks of a neighboring town have paraded the market with a remedy guaranteed to cure catarrh (whatever that is), with their photos attached. I know these men personally; they are otherwise nice, honorable gentlemen, but they are not physicians, and, as far as I know, have no such intentions; and if they are going to prescribe for the afflicted, they should be made to comply with the law like the rest of us do. It is a parady on the medical colleges, that they can not teach a man in four years to do what these rattle-heads can do in a few weeks, and for 25 cents, and a burlesque on the profession that they can't do it at all. "Suckers will bite," and they say there is one born every minute; still, it is your duty and mine to teach him better, imbue him with higher principles of intelligence, and put down the grafter.

I haven't much more patience with the proprietary man. It is an embarrassing reflection upon the medical teachings of our day, that the medical man doesn't acquire, neither during his collegiate training nor afterward, the knowledge of the remedies, nor the art of pharmacy to enable him to do his own compounding, instead of having to prescribe a ready-made, hand-me-down article, formulated and manufactured a thousand miles from his patient.

Some druggists in this State, and some in this town, put preparations on the market that they mix up themselves, put their names on the package, advertise and sell them to the public; then expect the physician to send them his prescriptions. This is what I call "gall." They have no more right to do this than you or I have to practice pharmacy without a license.

The law regulating the practice of medicine in our State is very good as far as it goes; that is, in some respects; in others, it is pernicious.

In my opinion, no one has a moral right to practice medicine who is not a graduate of a reputable medical college. Indeed, very few newly-made graduates are competent to assume the responsibilities of the risks of the health and happiness of the people. Those who would, should not be permitted to do so until they are fully prepared. Such facilities are now more than ample. The schools should be required to give them all sufficient instruction, clinical and didactic, before they are sent out to take their places at the bedside. Those who desire to enter the schools should be required to possess a good English education and some of the classics. This is put down now as a requirement by most of the schools, but seldom enforced; at least I have never seen one turned away.

I know physicians who can not write legibly, can not write a prescription correctly, not because of any mechanical inability, but because of the lack of a preliminary education; hence they are incompetent to solve the great problems that daily confront the medical man. Druggists very often call my attention to prescriptions sent into their pharmacies showing a woeful ignorance of the principles of prescription writing and compounding, and the want of elementary training.

A young man of this kind approached me the other day inquiring the cost of the required course. He is worthy in every respect, but has not the proper appreciation of the vocation to which he aspires. Let's discourage any but educated young men of sterling worth from entering our schools; tell them that the medical profession is not a dumping ground for those who are tired of the farm or have failed in business; that it is not easy work, not a permanent paying job; but, instead, it is a very expensive and laborious occupation; that to succeed it is, as Dr. Osler has said, "work, work, work," requiring the brainiest men, the keenest intellect, the profoundest reasoning, the deepest logic and the most untiring efforts, associated with an impelling desire to labor for the good of our fellow-man, to assuage human suffering, promote happiness; in a word, "to imitate the goodness of the Father of all men"; and that, with us, "merit constitutes the great title to our privileges."

I think that the new law is very unjust in not permitting the board to grant certificates to graduates who are practicing under the old. Probably those who are doing the bulk of the work graduated prior to 1891. [They are exempt; do not require a license. See Sec. 8 of the Medical Practice Act.—Editor.] They are those who have labored so faithfully and so long to elevate the standard and insist on better legislation; yet they are not permitted to enjoy the same rights and privileges as those who have graduated since. This is class legislation; a reflection on every old practitioner in the State, an insult to his alma mater, and a disregard for his long years of laborious efforts to keep its colors from trailing in the dust. In view of the coming law of reciprocity, it is an outrage. Every one should work against such legislation and its advocates.

I am opposed to midwives who are not graduates in medicine being granted license under any circumstances. This branch of our art is most sacred in its realm; often the most difficult and dangerous, requiring the most skillful manipulations, always the most thorough physiological and pathological acquaintance, with yet many unsolved problems. It is a wanton lack of chivalry, to say the least, that we permit such unwarranted disregard of care during the most important period of woman's existence.

There should be some law regulating the specialists. Some one not long ago said, that none but past masters in medicine should be allowed to announce himself a specialist; thoroughly prepared by long experience and special training, present himself before a competent and special board created by law, and if found efficient, granted a special license to practice his chosen specialty. This would thin down the ranks of the great horde of quacks whose caricatures of human physiognomies adorn our metropolitan dailies, save annually many thousand dollars to the worthy physician, as well as to the lugubrious sucker. I have known young men in their first year of practice, and some of them under-graduates, announce themselves as specialists in such and such branches. This is wrong. The public don't know all this; seemingly don't care.

In regard to the Osteopaths and Christian Scientists, I have this to say: No one should be allowed to practice the healing art who is not a graduate of a reputable medical college and having passed a satisfactory examination by the proper board.

Now, gentlemen, these are a few of the existing conditions and evils as I see them. Ours has been called a "noble profession, but a poor trade." Therefore it is of paramount importance that we recognize the full scope of the new regime that we have accepted from the A. M. A. The higher divisions of our associations expect the county societies to select the material for the great medical fraternity. To bestir local enthusiasm, get close to the people, in touch with all the physicians, explore the local as well as general needs. We are the "folks" of the medical brotherhood; not the cap sheaf, but the mud sill upon which the superstructure must be reared. If, therefore, our assets are to be valuable, we must dispose of our shelf-worn goods, renew our old paper, and liquidate our antiquated claims.

Take more journals and better ones; read them oftener and closer; take more post-graduate courses and longer, and live the lives that comport most nearly with the responsibilities that we have voluntarily assumed.

In turn, we must demand that those who poise above the fogs do not overstep the proprieties by which they would measure us. That they to whom are entrusted matters judiciary should hear the demands of those upon whom rests the integrity and the perpetuity of the organization. Then we may hope for the return of the waning pride of our art, an educated constituency, loyal in purpose, clean in action, imbued with principles of lofty Americanism, the ideal of humanitarianism, clothed in chastity, crowned with nobility commensurate with this great commonwealth of ours, the pride of the realm; charlatanry abolished, pretenders undone, testimonials forgotten, an honor and not a ridicule to become a medical student or to join a medical society.

